# Pulmonary Adenocarcinoma Occurring 5 Years after Resection of a Primary Pancreatic Adenocarcinoma: A Relevant Differential Diagnosis

**DOI:** 10.1155/2014/841907

**Published:** 2014-02-23

**Authors:** R. F. Falkenstern-Ge, M. Wohlleber, M. Kimmich, K. Huettl, G. Friedel, G. Ott, M. Kohlhäufl

**Affiliations:** ^1^Division of Pulmonology, Klinik Schillerhoehe, Center for Pulmonology and Thoracic Surgery, Teaching Hospital of the University of Tuebingen, Solitude Straße 18, Gerlingen, 70839 Stuttgart, Germany; ^2^Division of Clinical Pathology, Robert Bosch Krankenhaus, Teaching Hospital of the University of Tuebingen, Auerbachstraße 110, 70376 Stuttgart, Germany; ^3^Division of Thoracic Surgery, Klinik Schillerhoehe, Center for Pulmonology and Thoracic Surgery, Teaching Hospital of the University of Tuebingen, Solitude Straße 18, Gerlingen, 70839 Stuttgart, Germany

## Abstract

Ductal adenocarcinoma of the pancreas is a lethal disease. Surgical extirpation only offers the slim chance for long-term survival in localized disease. We report on a 73 year old female patient who initially underwent successful resection of pancreatic adenocarcinoma in May 2005. She was treated with adjuvant chemotherapy with gemcitabine. In October 2010 the patient noticed increasing dyspnea with haemoptysis. She was soon referred to our center. After the diagnosis of pulmonary adenocarcinoma with widespread metastasis, she was treated with systemic chemotherapy. For a period of next three years, she was treated with different chemotherapy regimens due to repeated episodes of tumor progression. To the best of our knowledge after reviewing the literature, this case represents an unusually clinical course with metachronous pulmonary adenocarcinoma arising after treatment of a primary pancreatic cancer after a long latency period.

## 1. Main Article

**Figure 1:**
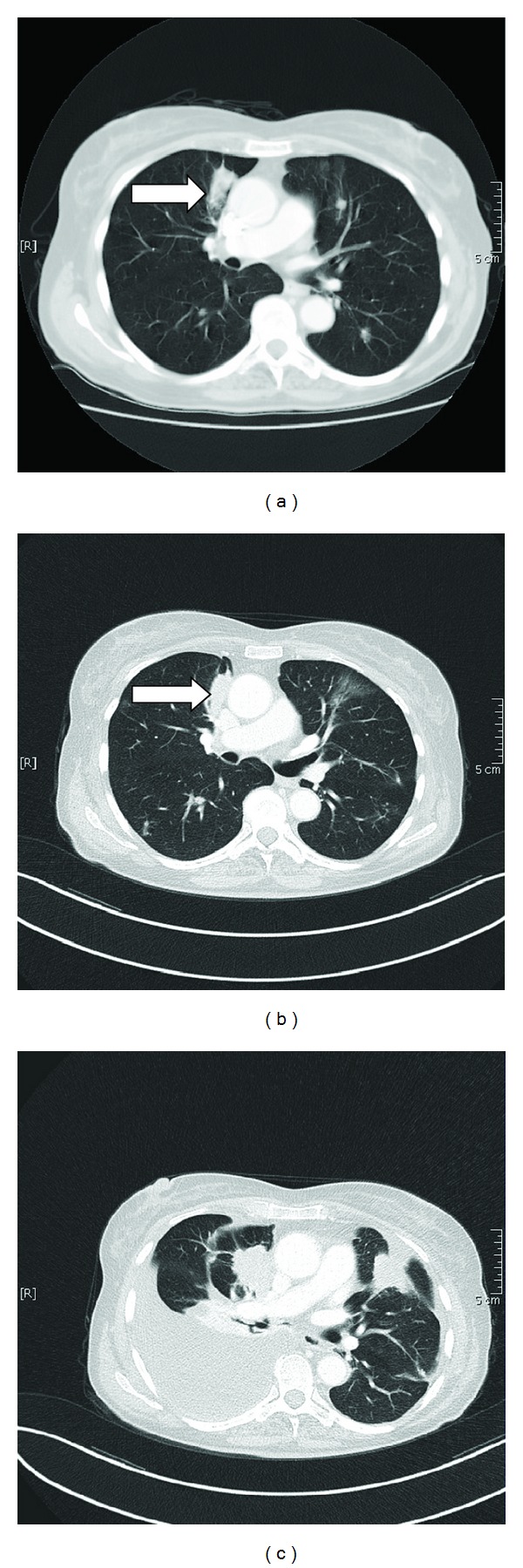
Contrast-enhanced tomography revealed a major pulmonary mass within the right upper lobe (arrow) (a). Stable disease was achieved under first line therapy with carboplatin and pemetrexed (b). CT-evaluation revealed tumor progression of the pulmonary mass and metastases, huge pleural effusion due to pleural carcinosis of the right side (c).

The patient had undergone distal pancreatectomy with portal vein resection for pancreatic body cancer in early May 2005. At the time of pancreatectomy, the cancer was confined to the organ and had not invaded the portal-splenic vein junction. To ensure an adequate surgical margin, subtotal distal pancreatectomy was performed. Histopathological examination revealed an invasive well-differentiated ductal adenocarcinoma of the pancreas. Adjuvant chemotherapy with gemcitabine was administered. All follow-up reevaluations showed no signs of tumor recurrence, and external laboratory tests did not show elevated CA 19-9 levels. In October 2010, she was referred to our center due to the increasing dyspnea with haemoptysis. Contrast-enhanced tomography revealed a major pulmonary mass in the right upper lobe with contralateral metastases (Figure 1(a)). Macroscopic histologic workup revealed tumor nodule measured 1.3 cm in maximum diameter and it was sharply demarcated. Histological examination revealed a well-differentiated adenocarcinoma with lepidic, papillary, and acinar growth patterns and intracellular and extracellular mucin production. A curative tumor resection was not feasible. First line systemic chemotherapy with carboplatin and pemetrexed was initiated. After five months, stable disease was achieved (Figure 1(b)).

During the next 3 years, different chemotherapy agents were required due to repeated episodes of tumor progression. After the second/third and fourth line therapy including erlotinib, gemcitabine, and vinorelbine, systemic chemotherapy was finally stopped due to low overall performance and the declining clinical condition of the patient. The most recent contrast-enhanced thoracic tomography revealed severe tumor progression of the pulmonary mass and metastases (Figure 1(c)). In order to optimize current supportive care, we inserted drainage pleurX-catheter due to the huge pleural effusion with pleural carcinosis of the right lung.

**Figure 2:**
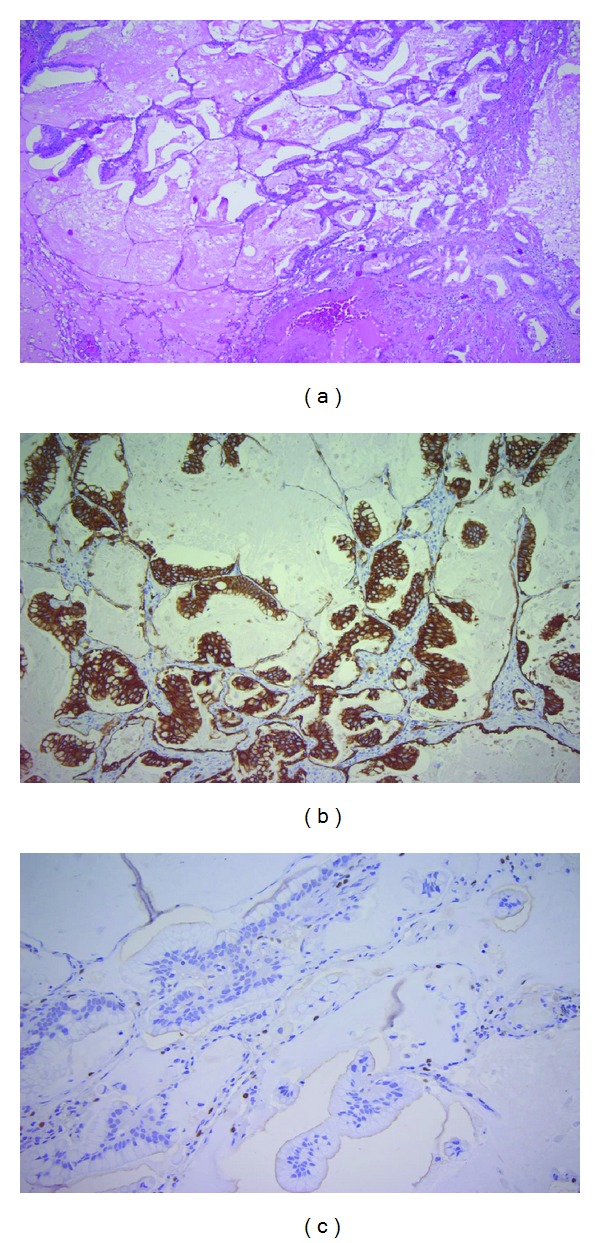
(a) HE-staining of the tumor clearly demonstrates a lepidic growth pattern (×50). (b) The tumor cells display a strong expression of CK7. (c) The tumor cells are negative for TTF-1 on immunohistochemistry (note positive internal control).

**Figure 3:**
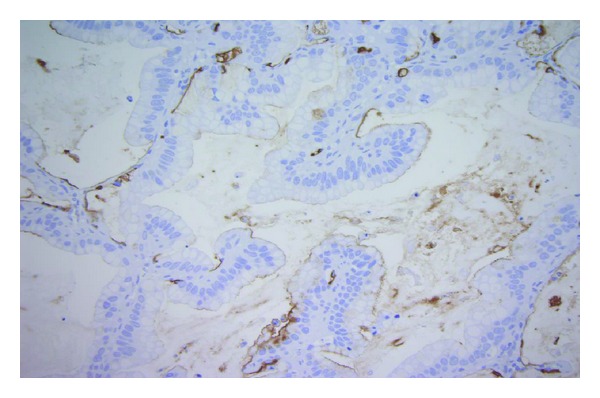
Anti-MUC1 staining shows a nonspecific weak partial membranous reaction in the tumor cells.

The tumor cells showed a strong expression of CK7, but no positive reactions for CK20, NapsinA, TTF-1, or MUC2 on immunohistochemistry (Figures 2(a)–2(c)). Notably, anti-MUC1 staining displayed a nonspecific weak partial membranous reaction in the tumor cells (Figure 3). Most primary mucinous adenocarcinoms of the lung neither express TTF-1 nor NapsinA, and therefore were not helpful markers in the present case. Considering the previous diagnosis of a moderately differentiated adenocarcinoma of the pancreas with “classical” ductal growth, the conventional morphology of the pulmonary tumor, especially the in part lepidic growth pattern, favored the diagnosis of a primary mucinous adenocarcinoma of the lung.

## 2. Discussion

We report a patient who successfully underwent Whipple pancreatectomy for pancreatic body cancer in early May 2005. In October 2010, she was diagnosed with metachronous pulmonary adenocarcinoma.

Schwarze et al. and colleagues described 3 patients with metachronous cancers of the lung and pancreas. In those cases, the patients were reported to develop the secondary cancer after 16–66 months [1]. The time frame of our patient with 65 months represents one of the longest intervals documented in patients, who developed late metachronous secondary lung carcinoma after pancreatic cancer.

The prognosis of patients with locally advanced pancreatic cancer is extremely dismal. Only few patients survive for longer periods of time, even if treated by pancreatectomy combined with blood vessel resection, extended lymph node dissection, and adjuvant therapy such as chemotherapy and radiation therapy [2, 3].

Initially, the pulmonary tumor was suspected to represent late metastasis of the previously resected pancreatic carcinoma. The incidence of pulmonary metastasis in that cancer is relatively low with only a percentage of 6.4% reported before [4, 5]. In our patient, the initial pancreaticoduodenectomy was performed as a curative intent and the lymph nodes were free of metastasis. All follow-up evaluations were performed within a regular time frame. No tumor recurrence was detected.

On the other hand, survival after Whipple's surgery is often short, and median overall survival times of 23.9 months were given in the literature for patients with pancreatic cancer that underwent resection [6]. We suppose that our observation has clinical significance as it suggests that contrary to current practice, surveillance of patients also beyond 5 years after pancreatic resections might be important to pick up those patients who fall into this categorical group. This case represents the first documentation of such a long latency between a primary pancreatic carcinoma and a secondary metachronous pulmonary adenocarcinoma.

Gemcitabine has been the standard treatment modality for more than 15 years for advanced pancreatic cancer. New combination chemotherapy regimens (e.g., FOLFIRINOX, nab-paclitaxel plus gemcitabine) achieved a significant survival benefit compared to gemcitabine alone [7]. Our patient also received the standard adjuvant chemotherapy with gemcitabine after the tumor resection 5 years ago.

The synergistic activity of pemetrexed with platinum agents in non-small cell lung cancer (NSCLC) and the renal safety of carboplatin suggest a very balanced benefit/risk profile for this combination in elderly patients. A multicenter single-arm phase II Study from Gervais et al. suggested that the combination of pemetrexed-carboplatin could be a valuable treatment option in elderly patients. Neutropenia remained the most common toxicity. Stable disease rate was achieved at 42.9%. Grade 3/4 toxicities related to study drugs were asthenia 16.1%, anorexia 4.8%, diarrhea 3.2%, neutropenia 51.6%, leucopenia 30.7%, thrombocytopenia 29%, and anemia 19.4%. In advanced NSCLC, pemetrexed use is restricted to nonsquamous histology [8].

In conclusion, we report on a patient who survived two different epithelial cancers for an unusually long period of more than 8 years. The time interval between the two cancers represents extreme long latency interval in a documented patient who was diagnosed with late metachronous pulmonary adenocarcinoma after resection of a primary pancreatic cancer.
